# Anti-Obesity Effect of Chlorin e6-Mediated Photodynamic Therapy on Mice with High-Fat-Diet-Induced Obesity

**DOI:** 10.3390/ph16071053

**Published:** 2023-07-24

**Authors:** Rajeev Shrestha, Pallavi Gurung, Junmo Lim, Til Bahadur Thapa Magar, Cheong-Wun Kim, Hak Yong Lee, Yong-Wan Kim

**Affiliations:** 1 Dongsung Cancer Center, Dongsung Biopharmaceutical, Daegu 41061, Republic of Korea; 2 INVIVO Co., Ltd., Nonsan 32992, Republic of Korea

**Keywords:** obesity, chlorin e6, photodynamic therapy, leptin, adiponectin

## Abstract

This study aimed to evaluate the efficacy of Chlorin e6 (Ce6)-based photodynamic therapy (PDT) for anti-obesity activities in high-fat-diet (HFD)-induced obesity mouse models. We induced obesity in C57BL/6 mice by HFD and administered Ce6 (2.5 or 5 mg/kg) orally with 3 h of incubation. The mice were then exposed to light of high fluence rate (4.96 mW/cm^2^) or low fluence rate (2.56 mW/cm^2^) in the designed LED mouse chamber 2–3 days a week for up to 8 weeks. The study also analyzed the pharmacokinetics and optimization of the drug by evaluating the absorption, distribution, metabolism, and excretion (ADME) of Ce6 in the rat models. Both low doses (2.5 mg/kg) and high doses (5 mg/kg) of Ce6 with high irradiation dose showed better anti-obesity effects than other groups with decreased body weight. The lipid accumulation in the liver and adipocyte size in epididymal adipose tissues were found to be decreased by Ce6-PDT in comparison to vehicle-treated HFD groups. We also observed increased levels of the lipidomic biomarkers, such as leptin and LDL cholesterol, while observing decreasing levels of total cholesterol and adiponectin in the Ce6-PDT-treated mice. These findings may provide valuable insight into Ce6-PDT as an alternative and non-invasive therapeutic methodology for obesity and obesity-related diseases.

## 1. Introduction

Over the past decade, obesity has become a significant concern due to its increased risk of major health problems such as type II diabetes, hypertension, cardiovascular disease, stroke, sleep apnea, and cancer [1,2]. Obesity is primarily caused by a high-calorie diet and a lack of physical activity [3]. Adipose tissue is the primary organ responsible for storing fat in the body, and an abnormal amount of fat deposits leads to obesity [4]. Adipose tissue plays an important role in metabolic regulation and physiological homeostasis through the secretion of multiple hormones, including adipokines such as leptin and adiponectin [5,6]. The adipocytokine leptin is related to energy storage in both white adipose tissue and plasma [7]. It has been observed that leptin increases in obese individuals and drops during periods of fasting [8,9]. On the other hand, adiponectin is a plasma protein that decreases when obesity occurs and correlates with insulin sensitivity [7,10]. Furthermore, weight loss can cause a substantial increase in plasma adiponectin levels [11]. Therefore, these biomarkers for obesity are known to have an influence on several metabolic activities involved in controlling body weight and energy expenditure [12].

In recent years, studies have shown that obesity-induced non-alcoholic fatty liver diseases (NAFLDs) can lead to increased levels of liver enzymes such as aspartate aminotransferase (AST) and alanine aminotransferase (ALT) and pose a significant risk of metabolic disorders and mortality [13]. AST and ALT are liver enzymes that assist in the breakdown of proteins and are released into the bloodstream when the liver is damaged or inflamed. High levels of AST and ALT indicate hepatic dysfunction [14,15]. In addition, obesity results in cardiovascular disease due to imbalances of lipoproteins or lipids such as triglycerides (TG), total cholesterol (TC), high-density lipoprotein (HDL), low-density lipoprotein (LDL), and very-low-density lipoprotein (VLDL) [16,17].

The main approaches for confronting the obesity problem are lifestyle intervention, pharmacotherapy, and bariatric surgery [18,19]. Achieving long-term weight loss often requires more significant lifestyle modifications than simple diet and exercise. However, many research studies have shown that it can be challenging to maintain such modifications over an extended period [20]. In this regard, photodynamic therapy (PDT) has become an alternative, non-invasive therapeutic modality for the treatment of obesity [21,22,23]. The replication and differentiation of pre-adipocytes may increase the number of adipocytes. Pre-adipocyte and adipocyte apoptosis cause adipocyte numbers to decline. Previous studies have shown that PDT with DH-I-180-3 induces cell death in both undifferentiated and differentiated 3T3-L1 cells effectively [24]. By using specific photosensitizers (PSs), irradiating light at a range of 600–800 nm results in the destruction of targeted preadipocytes and adipocyte cells in the body via apoptosis caused by the resultant stress on fat cells or restricted cell necrosis. However, according to the research, a tight control of apoptosis induction is required due to the negative metabolic effects of fat mass loss, such as lipodystrophic disorders; therefore, targeted activation of adipocyte apoptosis seems to be important [25]. Such adipocyte death results in a decrease in the fat layer [26,27]. Furthermore, in PDT, the adipocytes are emulsified by light irradiation through a combination of chemical and thermal phenomena, breaking the cell walls and releasing the cell fluid. Such a procedure guarantees secure and long-lasting fat removal, lessens injury to the treatment area, and speeds up the healing process [26]. In particular, liposomal formulations of photosensitizers or the systemic administration of drugs followed by photoactivation with a given wavelength and light energy are known to selectively target subcutaneous adipose tissue [25]. In recent years, PDT with methylene blue, veteporfin, and indocyanine green has been known to reduce fat [27,28,29]. 

After receiving the remarkable results of Ce6-mediated PDT for cancer treatments [30,31,32], we introduced Ce6-PDT for the first time for its anti-obesity activities in vivo. In an earlier study, we demonstrated the potential inhibitory roles of Ce6-PDT on adipogenesis and lipogenesis in 3T3-L1 cells [33]. Herein, we have analyzed the pharmacokinetics and optimization of drug therapy in obese mouse models by evaluating the absorption, distribution, metabolism, and excretion (ADME) of Ce6. Moreover, we have designed the LED mice chamber for Ce6-PDT treatment in mice fed with normal diets or high-fat diets. Similarly, the liver and epididymal weights, leptin and adiponectin levels, toxicity markers of the liver and kidney (ratio of AST/ALT, BUN, and creatinine), along with biochemical serums (HDL, LDL, TG, TC, and VLDL) were also studied for a better understanding of Ce6-PDT effects in HFD-induced obese mouse models.

## 2. Results

### 2.1. Bio Distribution of Ce6

A pharmacokinetic test (PK) was validated using mice and rats to comprehend the connection between drug dose and drug concentration in the body. A total of 5 mg/kg of Ce6 was orally administered to the normal mouse, normal rat, and fat mouse (Table 1). Later, the concentration of Ce6 in the blood serum was studied at different time periods. The maximum concentration of Ce6 in the blood (16,685.4 ng/mL) of the normal mouse was found at 0.5 h after oral administration. However, in the cases of the rats and fat mice, maximum concentrations (241.4 and 129.9 ng/mL, respectively) were observed 4 h after administration.

### 2.2. ADME Evaluation

ADME experiments were performed to assess the liver microsomal and plasma stability of Ce6 in rats and to predict Ce6 interactions in human liver (Table 2). The metabolic stability of Ce6 was analyzed by the administration of Ce6 (1 µM) to rat liver microsomes. After 30 min., the result revealed that the metabolic stability of Ce6 in the liver tissue of the rat was 99.6% and 85.4% in the dog relative to the unreacted control. On the other hand, when Ce6 (10 µM) was examined in rat plasma, Ce6 plasma stability was found to be 42 and 45.3% at 30 and 120 min., respectively. Ce6 plasma stability at 120 min. was compared to the reference drugs like Procaine and Enalapril (Table 3). Moreover, to predict the drug interactions, the inhibitory ability of Ce6 for CYP isoenzyme, a major drug metabolizing enzyme in the human liver, was measured in terms of percentage of activity relative to the control without inhibitor. The result depicts that the inhibitory role of Ce6 in the CYP isoenzyme activity was in the range of 33–90% (Table 4). Ketoconazole was used as a reference drug to compare the inhibition of CYP activity by Ce6.

### 2.3. Preliminary Study of Ce6-PDT in an Obesity Mouse Model 1

**Figure 1:**
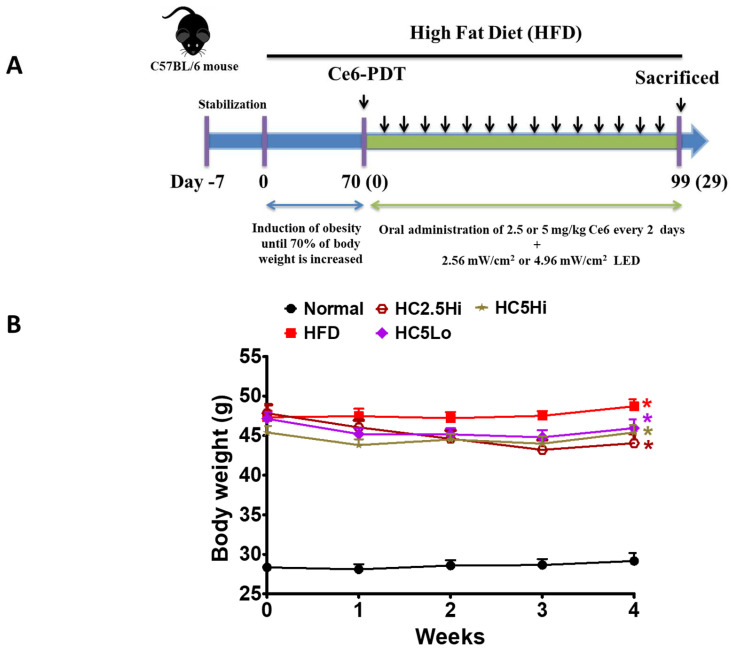
Effects of an oral dose of Ce6-PDT on an HFD-fed C57BL/6 mouse’s body weight (Model 1). (**A**). Experimental design and timeline of mouse model 1 with normal and HFD groups. The mice were fed a normal diet or a high-fat diet. Mice in vehicle-treated HFD (HFD) groups were administered orally with normal saline, while drug-treated groups were given 2.5 mg/kg of Ce6 with light of a high fluence rate (4.96 mW/cm^2^) of 660 nm (HC2.5Hi), 5 mg/kg of Ce6 with light of a high fluence rate (HC5Hi), and 5 mg/kg of Ce6 with light of a low fluence rate (2.56 mW/cm^2^) (HC5Lo) every 2 days, respectively. The normal-diet-fed group was given no treatment. (**B**). Changes in body weights of normal and HFD groups after induction of obesity. Data are expressed as mean ± SD (*n* = 4, Normal, HFD, HC2.5Hi, HC5Hi, and HC5Lo groups) * *p* < 0.01 compared with vehicle-treated control.

To evaluate the effect of Ce6-PDT on anti-obesity efficacy, we have used mouse model 1 as a preliminary experiment, wherein the mice were divided into two groups: mice with a normal diet and HFD groups (Figure 1). When the body weight of the mice increased by 70% with HFD (HFD containing 60% fat) in 10 weeks, we randomly assigned them to four groups: vehicle-treated HFD only; high-fat diet with Ce6, 2.5 mg/kg, and light of a high fluence rate (HC2.5Hi); high-fat diet with Ce6, 5 mg/kg, and light of a low fluence rate (HC5Lo); and high-fat diet with Ce6, 5 mg/kg, and light of a high fluence rate (HC5Hi) (Table 3). The mice were then administered an oral dose of Ce6 (2.5 or 5 mg/kg), and 3 h [29] later, PDT was performed using a 660 nm laser irradiation (2.56 mW/cm^2^ and 4.96 mW/cm^2^), while vehicle-treated HFD mice were administered with normal saline. PDT was repeated every 2 days for up to 4 weeks. We found that Ce6-PDT suppressed the body weight gain of HFD-fed mice (HC2.5Hi, HC5Lo, and HC5Hi) until the end of the treatment (the 28th day), in comparison to the normal mice. Among them, HFD-fed mice treated with 2.5 mg/kg or 5 mg/kg of Ce6 with a high 4.96 mW/cm^2^ of PDT showed good anti-obesity effects.

### 2.4. Ce6 Anti-Obesity Efficacy Evaluation in Mouse Model 2

**Figure 2:**
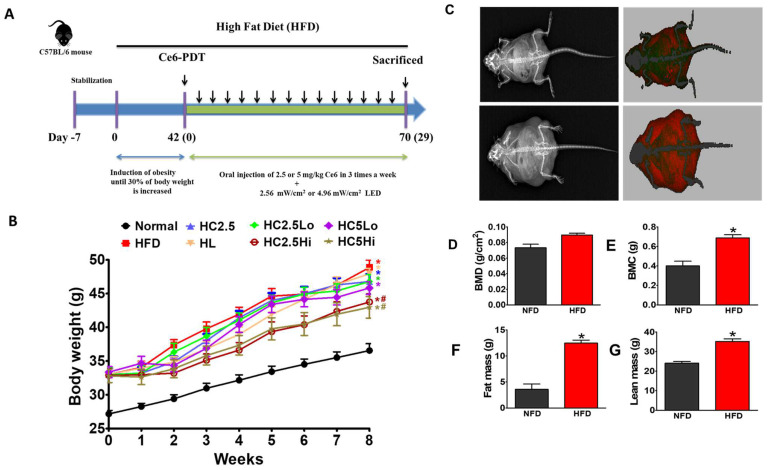
Treatment of HFD-fed mice with Ce6-PDT reverses obesity. (**A**) Experimental timeline for obesity study set 2 (8 experimental groups). The mice were provided with either a normal diet or a high-fat diet. Mice in vehicle-treated HFD (HFD) groups were administered orally with normal saline, while drug-treated groups were given 2.5 mg/kg of Ce6 with light of a low fluence rate (2.56 mW/cm^2^) of 660 nm (HC2.5Lo), 2.5 mg/kg of Ce6 with light of a high fluence rate (4.96 mW/cm^2^) (HC2.5Hi), 5 mg/kg of Ce6 with light of a high fluence rate (HC5Hi), and 5 mg/kg of Ce6 with light of a low fluence rate (HC5Lo) every 2 days, respectively. HL and HC2.5 were considered light-only-treated and Ce6-only-treated control groups. Mice in vehicle-treated HFD groups were administered orally with normal saline. The normal diet group of mice was not exposed to any treatment. (**B**). Changes in body weight for different groups of mice. Data are expressed as mean ± SD (*n* = 7). * *p* < 0.05 compared with the control group. ^#^ *p* < 0.05 compared with the HFD-only-treated group. (**C**) Representative X-ray (left) and DEXA (right) images (**D**) of bone mineral density (BMD, g/cm^2^), (**E**) bone mineral content (BMC, g), (**F**) fat mass (g), and (**G**) lean mass (g) of the normal and HFD-fed mice, respectively. Data are expressed as mean ± SD (*n* = 7, Normal, HFD, HL, HC2.5, HC2.5Lo, HC2.5Hi, HC5Lo, and HC5Hi). * *p* < 0.05 compared with the normal mice.

After determining the efficacy of Ce6-PDT with the preliminary anti-obesity experiment in model 1, we continued our investigation with model 2 by adding two extra controls, namely HC2.5 (high-fat-diet mouse with Ce6 only, 2.5 mg/kg) and HL (high-fat-diet mouse with light only). Model 2 had eight groups of mice in different conditions, i.e., normal, vehicle-treated HFD (control 1), HC2.5, HL, HC2.5Lo, HC2.5Hi, HC5Lo, and HC5Hi (Table 4) (Figure 2A,B). Major features of the model 1 and model 2 experiments have been listed in Table S2 (Supplementary Materials). The experimental groups of mice were fed either a normal diet or a high-fat diet according to the experimental procedure shown in the timeline. The daily intake of food and water during the experimental procedure has been provided in Figure S1, Supplementary Materials. Mice were administered 2.5 or 5 mg/kg of Ce6 by oral gavage for 3 h followed by PDT (2.56 mW/cm^2^ and 4.96 mW/cm^2^, respectively, from 660 nm lasers). The weights of the mice were evaluated once a week for 8 weeks. HFD mice weighed significantly more than mice fed with a normal diet. At the end of the experiment, mice in the HC2.5Hi and HC5Hi groups showed significant reductions in body weight of 22.3% and 26.6%, respectively, in comparison with the HFD mice group. Similarly, the HC5Lo group also showed moderate anti-obesity effects, while the HC2.5Lo group showed the least effects compared to the vehicle-treated HFD groups. A DEXA scan (Figure 2C–G) confirmed that, upon administration of a high-fat diet, the BMD was increased in comparison to normal mice (Figure 2D). After the HFD, bone mineral content (BMC) (Figure 2E), total fat mass (Figure 2F), and lean mass (Figure 2G) were significantly increased in HFD-fed mice in comparison to normal mice. The second experiment, therefore, correlates with the first preliminary experiment as it demonstrates that the HC2.5Hi and HC5Hi mouse groups showed the best Ce6-PDT-induced anti-obesity effect over 4 weeks. However, in 8 weeks, the HC5Hi mice group demonstrated a slightly higher anti-obesity effect than the HC2.5Hi group.

### 2.5. Morphological Changes in Hepatocytes and Epididymal Adipocytes

**Figure 3:**
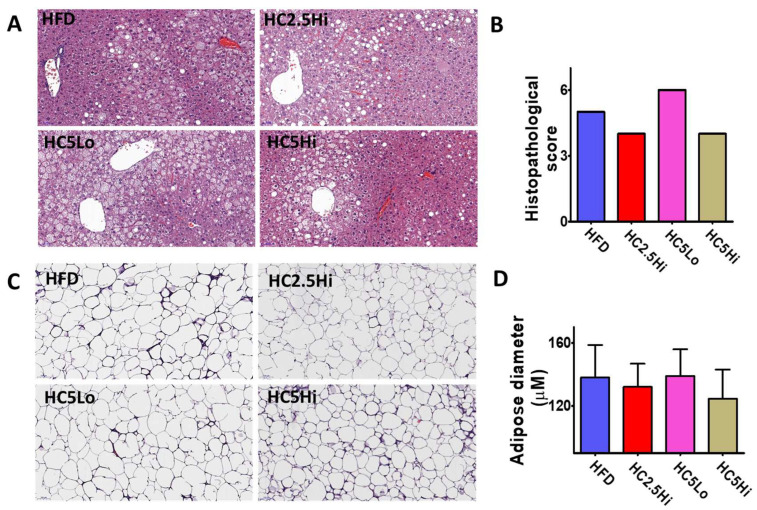
Effects of Ce6-PDT on hepatocytes and epididymal adipose tissue: (**A**) H&E stained sections of liver (×200) and (**B**) histopathological score in vehicle-treated HFD, HC2.5Hi, HC5Lo, and HC5Hi groups. The data are presented as the mean of a single mouse. (**C**) Image of epididymal adipose tissue (×200) (**D**) and adipose diameter in HFD, HC2.5Hi, HC5Lo, and HC5Hi groups. Data were represented as mean ± SD (*n* = 3, HFD, HC2.5Hi, HC5Lo, and HC5Hi).

This study also evaluated the degree of steatosis, inflammation, and hepatocellular hypertrophy according to the general non-alcoholic fatty liver disease (NAFLD) score [34,35] in the hepatocytes of vehicle-treated HFD and Ce6-PDT-treated HFD mice (Figure 3A,B, Table S3). As expected, mice fed HFD for 15 days displayed enlarged livers in comparison to the groups that were treated with Ce6-PDT. Examination of H&E-stained sections of the liver indicated that HFD-fed mice showed extensive microvascular steatosis, hepatocellular hypertrophy, fibrosis, and inflammation, showing an increase in the NAFLD activity score. However, histopathological examination of liver sections confirmed reduced steatosis, hepatocellular hypertrophy, and inflammation, resulting in a lowered NAFLD activity score for HC2.5Hi and HC5Hi groups, which decreased with Ce6-PDT treatment.

Furthermore, hematoxylin and eosin (H&E) staining of the epididymal adipose tissues revealed adipocyte hypertrophy in HFD-fed mice (Figure 3C). The small number of cells per field is observed in HFD-fed mice, suggesting increased adipocyte size. Interestingly, the adipocyte diameter was slightly reduced in the HC2.5Hi and HC5Hi groups compared to the cells in the HFD diet group (Figure 3C,D).

### 2.6. Evaluation of Organ Weights

**Figure 4:**
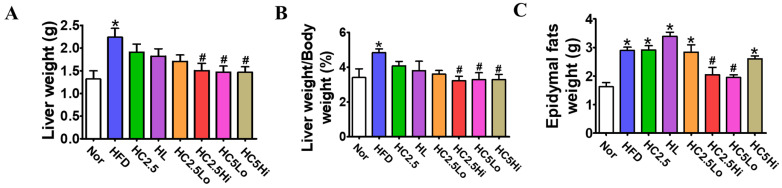
Ce6-PDT decreases liver and epididymal adipose tissue weight in an obese mouse model 2. Changes in weights of (**A**) liver and (**B**) epididymal fats after the sacrifice of mice; (**C**) Liver weight/body weight ratio (%). Data are expressed as mean ± SD (*n* = 7, Normal, HFD, HL, HC2.5, HC2.5Lo, HC2.5Hi, HC5Lo, and HC5Hi). * *p* < 0.05 compared with the control group. ^#^ *p* < 0.05, compared with the HFD-only-treated group.

Since Ce6-PDT suppressed HFD-induced weight gain, we measured individual tissue weight (Figure 4A,B). The weight of internal organs such as the liver and epididymis is responsible for the increase in body weight. Similar to body weight, liver and epididymal weights were also significantly increased in vehicle-treated HFD mice after 6 weeks of HFD feeding. To investigate the effects of Ce6-PDT on liver and epididymis weights in HFD mice, livers and epididymis were removed from sacrificed experimental mice and liver weights, liver weight-to-body weight ratios, and epididymis weights were measured (Figure 4). The results indicate that the fatty weight of the liver in the Ce6-PDT-treated groups HC2.5Hi (2.19 ± 0.24 g), HC5Lo (2.05 ± 0.11 g), and HC5Hi (2.84 ± 0.12 g) was significantly decreased compared to the normal and HC2.5Lo groups (Figure 4A). In addition, in the case of epididymal fats, the HC5Lo and HC2.5Hi significantly decreased fat weights (Figure 4B). However, Ce6 (5 mg/kg) with light of a high fluence rate showed a lesser reduction, while HC2.5Lo showed no effect on the HFD-mediated elevation of epididymal fats.

### 2.7. Determination of Leptin and Adiponectin by ELISA

**Figure 5:**
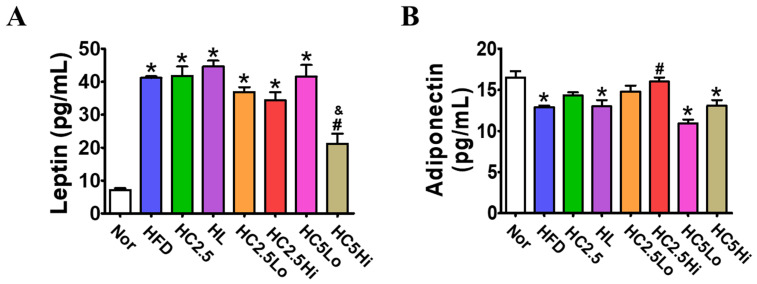
Serum leptin and adiponectin levels in obesity mouse model 2. Changes in (**A**) leptin level and (**B**) adiponectin level in the serum after mice were sacrificed. Leptin and adiponectin levels were determined using a mouse ELISA kit assay. Data are expressed as mean ± SD (*n* = 7, Normal, HFD, HL, HC2.5, HC2.5Lo, HC2.5Hi, HC5Lo, and HC5Hi). * *p* < 0.05 compared with the control group. ^#^ *p* < 0.05 compared with the HFD-only-treated group. ^&^ *p* < 0.05 compared with the H5Lo group.

To assess the potential anti-obesity effects of Ce6-PDT, obesity markers such as leptin and adiponectin were also measured in the mice’s serum (Figure 5). Serum leptin was significantly increased in the vehicle-treated HFD group but decreased in the Ce6-PDT-treated group compared with the normal treatment group. Serum leptin levels were decreased dramatically by the HC5Hi group in the greatest amount (22 ± 1.2 ng/mL), while other groups (HC2.5Lo, HC2.5Hi, and HC5Lo) showed declines in comparison to HFD-fed mice. Leptin reduction with HC5Lo and HC5Hi differed significantly, showing that an irradiation time of 10 min. was more effective than an irradiation time of 5 min. In contrast, the HC2.5Hi and HC2.5Lo groups restored the level of adiponectin decreased by HFD treatment. However, HC5Lo and HC5Hi showed no changes in adiponectin levels. These results indicate that Ce6-PDT is effective in enhancing adiponectin and suppressing leptin.

### 2.8. Serum Lipid Profile

**Figure 6:**
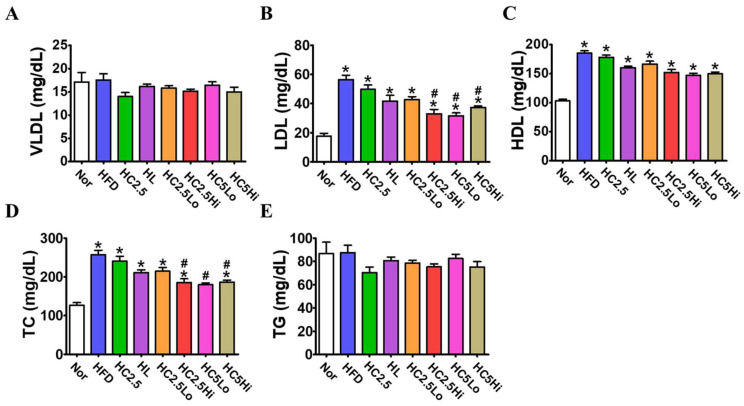
Serum levels of VLDL, LDL, HDL, TC, and TG in obese mice model 2. Changes in (**A**) VLDL, (**B**) LDL, (**C**) HDL, (**D**) TC, and (**E**) TG levels in the serum after mice were sacrificed. Data are expressed as mean ± SD (*n* = 7, Normal, HFD, HL, HC2.5, HC2.5Lo, HC2.5Hi, HC5Lo, and HC5Hi). * *p* < 0.05 compared with the control group. ^#^ *p* < 0.05 compared with the HFD-only-treated group.

The serum lipid profiles of the obese mice were greatly affected by the HFD diet. In the vehicle-treated HFD-fed mice and Ce6-PDT-treated mice, there were no significant changes in TG and VLDL values in comparison to normal mice (Figure 6). The LDL level of HFD-treated mice was increased in relation to the normal mice; however, it was reduced considerably in the HC2.5Hi, HC5Lo, and HC5Hi groups. HDL is considered an important factor in reducing lipid levels in the blood. Our results reveal that the HDL levels in the Ce6-PDT-treated groups (HC2.5Lo, HC2.5Hi, HC5Lo, and HC5Hi, respectively) were similar. 

Since the liver and kidneys are crucial in the metabolism of drugs, these organs prevent cells from being destroyed, although they can be seriously damaged in this process. Therefore, liver and kidney toxicity markers, aspartate aminotransferase/alanine transferase (AST/ALT), blood urea nitrogen (BUN), and creatinine, were measured in mice to assess the hepatotoxicity and renal toxicity of Ce6-PDT treatment (Figure S2, Supplementary Materials). It was confirmed that the ratio of AST/ALT was 1 or more in all liver values. In the case of BUN, all groups had levels that were lower than those of the normal group, indicating that there was no abnormality in the blood urea level with Ce6-PDT treatment. Additionally, the creatinine levels did not differ significantly from those of normal groups. Thus, it was established that Ce6-PDT had no adverse effect on kidney or liver function.

## 3. Discussion

Obesity is considered an epidemic that is rapidly spreading across the globe and causing a variety of metabolic diseases [1]. In recent years, PDT has become an alternative choice for individuals to reduce their weight amidst the hustle of life, besides various therapeutic treatments for obesity and its related metabolic disorders [26,27,28,29,30]. For the first time, the two different murine models of obesity investigated the pharmacokinetic properties and ADME studies as well as evaluated the anti-obesity effects of Ce6-PDT by using PDT chamber systems.

To determine the correlation between the dose of a drug and its concentration in the body, we found the half-life of Ce6-PVP (5 mg/kg) to be 0.5 h in the normal tissues of mice and 4 h in the normal rat or fat mice, respectively. The rapid accumulation of Ce6 within a short period of time and its fast drug clearance are advantageous for preventing drug accumulation and its toxicity [32,36]. The effects of drugs are mainly due to the metabolic changes in the drug and its ability to be absorbed into the body. The degree of drug metabolism in the liver microsomes can be accessed to predict the drug’s metabolic stability in vivo or its clearance [37]. The liver microsomal stability studies for Ce6 during 30 min. revealed a 99.6% rate in rats, which corresponds to the fact that Ce6 has comparatively higher stability in the liver than compounds such as verapamil, diazepam, etc. However, it is equivalent to stable compounds such as ceftriaxone, tenoxicam, etc. [38]. Similarly, during the screening stage of drug evaluation, factors such as its instability in the given conditions, plasma stability, and metabolic reactions are taken into account, as they can result in rapid clearance and a short half-life when administered in vivo [39]. Ce6 with rat plasma showed Ce6 stabilities of 42% and 45.3% at 30 and 120 min., respectively. The effectiveness of the drug was also further evaluated by measuring the CYP isoenzyme inhibition activity in the liver. Ce6-PDT showed the highest inhibitory effect on CYP2D6, followed by CYP2D9, CYP3A4, and CYP2C19. On the other hand, Ce6-PDT showed a modest increase in the activity of CYP1A2.

A high-fat diet has been associated with a faster increment in adiposity and induces obesity [40]. The researchers have been utilizing HFD intervention in rodent models of obesity with the aim of studying lipid metabolisms, metabolic disorders, and alternative ways of reducing body weight [41]. Previously, methylene blue (MB) as a photosensitizer embedded on an intragastric satiety-inducing device (ISD) and endoscopic-guided PDT were successful in lowering body weights in porcine models [27]. Our study on Ce6-PDT has also enabled the potential for significant weight loss. Additionally, Ce6-mediated PDT treatment effectively altered the morphological changes and showed a 60% reduction in lipid accumulation after 10 μM Ce6-mediated PDT in 3T3 L1 cells. This study also showed that Ce6-PDT impaired adipocyte differentiation and reduced lipid accumulation, which was a result of the inhibition of PPAR-γ, C/EBPα, β, δ, SREBP-1, FAS, and LPL expression through AMPK activation in 3T3 L1 cells [33]. Apart from that, Ce6 may remain for a longer period of time since areas of fatty tissue have fewer blood vessels and may exhibit superior efficacy. Studies have demonstrated that Ce6 and other photosensitizers become localized within cells in the cytoplasm and lipid droplets in adipocytes and preadipocytes [24,33]. However, further research is required to fully understand the mechanism of Ce6 accumulation in adipocytes. There are visceral and subcutaneous varieties of adiposity; however, Ce6-PDT targets visceral adiposity.

The anti-obesity effects of Ce6-PDT have never been explored in animal models. In the current study, an obesity model was performed using two separate sets of mice. Briefly, the preliminary set of mouse models included five groups: Normal, vehicle-treated HFD (control 1), HC2.5Hi, HC5Lo, and HC5Hi. In this study, the HFD mice gained their maximal body weight in 10 weeks, after which Ce6-PDT therapy was administered for an additional 4 weeks. Mice fed with HFD exhibited apparent weight gain (*p* > 0.05); compared with normal mice, the weight gain was reduced by 2.5 mg/kg and 5 mg/kg of Ce6 with light of a high fluence rate (4.96 mW/cm^2^) treatment. Then, we established a second mouse model to ascertain the anti-obesity benefits of Ce6-PDT which was performed in InVivo Co., Ltd. (Non-Clinical CRO, Nonsan, Republic of Korea). The second set of mouse models had eight groups: normal, vehicle-treated HFD (control 1), HC2.5 (control 2), HL (control 3), HC2.5Lo, HC2.5Hi, HC5Lo, and HC5Hi. Vehicle-treated HFD-fed mice had significantly (*p* < 0.05) more weight gain, liver weight, and epididymal fat compared to mice in the normal diet (NFD) group after 6 weeks. The phenotype of mice fed with HFD showed increased BMC, bone mineral density (BMD), total fat, and lean mass in contrast to normal mice. However, the treatment of HFD-fed mice with 5 mg/kg of Ce6 with light of high fluence rate (4.96 mW/cm^2^, 660 nm, 10 min.) significantly decreased body weight gain, liver weight, and epididymal fat compared to other groups. Treatment with 2.5 mg/kg of Ce6 and light of a high fluence rate also significantly attenuated weight gain and dramatically reduced liver weight, liver/body weight ratio, and epididymal fat. Therefore, the HC5Hi and HC2.5Hi groups showed marginally strong anti-obesity effects over the course of 8 weeks. Additionally, compared to other groups, HC5Lo and HC2.5Lo demonstrated the least anti-obesity efficacy. 

Excessive intake of HFD causes lipid accumulation in the liver and epididymis, which may be responsible for the increased body weight [42], and these effects were also observed in our study. As growth in the mass of adipose tissue and the number and size of fat cells are common characteristics of obesity, in our experiment, adipocyte size and histopathological score were shown to be enhanced in mice fed with HFD, but Ce6-PDT treatment reversed these effects in the HC5Hi and HC2.5Hi groups. These results suggest that Ce6-PDT effectively decreased the proliferation and differentiation of adipocytes. We also report that the administration of Ce6 in HFD mice did not induce liver and kidney damage, indicating that Ce6-PDT is non-toxic.

Leptin and adiponectin, the primary adipokines from adipocytes, are known to impact obesity and insulin sensitivity on a systemic level [43]. Therefore, these adipokines are also novel predictors for cardiometabolic and other chronic diseases [44]. Generally, a high-fat diet is known to up-regulate the leptin level and down-regulate the adiponectin level in obese mice [45]. In the current study, we found that Ce6-PDT treatment on HC2.5Hi groups resulted in a remarkable decrease in leptin levels by 16.5%, respectively, and an increase in adiponectin levels by 10.2%, respectively, when compared to HFD-fed mice. Moreover, the studies have revealed that the reduction in VLDL, LDL, triglycerides, and total cholesterol, as well as an increase in HDL levels, are directly associated with anti-obesity effects and cardiovascular diseases [46]. In this regard, our result shows a significant reduction in LDL and total cholesterol that had been increased by HFD in the HC2.5Hi, HC5Lo, and HC5Hi groups, respectively. However, no significant changes were detected in VLDL, HDL, or triglycerides in the Ce6-PDT-treated groups in comparison with the HFD group. Additionally, we discovered that a longer irradiation time with Ce6 resulted in better anti-obesity effects than a shorter irradiation time. A longer irradiation time must have allowed the Ce6 photosensitizer to penetrate deeper into the adipose tissue, allowing for a greater reduction in body weight and other obesity markers like leptin.

## 4. Materials and Methods

### 4.1. Ce6 and Its Sample Preparation

In the present work, we have used the Ce6 prepared in-house by the previously reported method [30]. The lyophilized powder of Ce6-PVP in the ratio of 1:1 (Phonozen^®^, Dongsung Biopharmaceutical, Seoul, Republic of Korea) was prepared, which was then dissolved in normal saline to make the desired concentration prior to oral administration. 

### 4.2. Preparation of Mouse Serum, Rat Serum, and Fat Mouse Serum

For the HPLC measurement, blood was collected from normal and obese mice orally administered 5 mg/kg of Ce6. Blood serum was extracted and examined to quantify the Ce6 concentration at different time periods. The serum (20 µL) was mixed with acetonitrile (80 µL), centrifuged at 150,000 rpm for 5 min, and LC–MS/MS was analyzed.

### 4.3. Sample Preparation for Microsomal Stability Assay

The evaluation of the metabolic stability of Ce6 was conducted by adding 1 µM of it to liver microsomes (Rat 0.5 mg/mL) in 0.1 M phosphate buffer at pH 7.4. The pre-incubation at 37 °C for 5 min. was followed by the addition of nicotinamide adenine dinucleotide phosphate (NADPH) regenerative system solution and a further incubation at 37 °C for 30 min. The reaction was terminated by adding an acetonitrile solution containing an internal standard (chlorpropamide). This mixture was centrifuged for 5 min. at 14,000 rpm, 4 °C, and the supernatant was injected into the LC–MS/MS system to analyze the substrate drug [47].

### 4.4. Sample Preparation for Ce6 Plasma Stability

Rat plasma with Ce6 (10 µM) was incubated at 37 °C in a tube at different time points (0, 30, and 120 min). An acetonitrile solution with an internal standard (chlopropamide, Sigma Aldrich, T9652, St. Louis, MO, USA) was added to the tube, vortexed for 5 min, and centrifuged (14,000 rpm, 4 °C) for 5 min. Subsequently, the supernatant was injected into an LC–MS/MS system to assess the plasma stability of Ce6 [38,48].

### 4.5. Sample Preparation for Inhibition of Cytochrome P450 (CYP) Isoenzyme Activity by Ce6

Human liver microsomes (0.25 mg/mL), 0.1 M phosphate-buffered solution (pH 7.4), substrate drug cocktail of 5 drug-metabolizing enzymes (Phenacetin (50 µM), Diclofenac (10 µM), S-mephenytoin (100 µM), Dextromethorphan (5 µM), and Midazolam (2.5 µM)), and Ce6 were added at a concentration of 0 and 10 µM and were pre-incubated at 37 °C for 5 min. NADPH generation system solution was added and incubated at 37 °C for 15 min. Then, an acetonitrile solution containing an internal standard (Terfenadine, Sigma Aldrich, T9652, St. Louis, MO, USA) was added to terminate the reaction, and it was centrifuged (14,000 rpm, 4 °C) for 5 min. The supernatant was injected into an LC–MS/MS system to simultaneously analyze the metabolites of the substrate drug, thereby evaluating the drug metabolizing enzyme inhibition ability of Ce6.

### 4.6. LC–MS/MS Analysis

Nexera XR system (Shimadzu, Kyoto, Japan) using a TSO vantage (Thermo) was used to analyze the amount of substrate remaining through the reaction. A kinetex C18 (2.1 × 100 mm, 2.6 µm) particle size; Phenomenox) was used as a HPLC column. The mobile phase was distilled water containing 0.1% formic acid (A) and acetonitrile containing 0.1% formic acid (B). The gradient program was 0–40% (B) in 0–1 min., 40–50% (B) in 1–4 min., 50–100% (B) in 4–4.1 min, and 100% (B) in 4.1–7 min. The injection volume of the sample was 2 µL with a flow rate of 0.3 mL/min. HPLC analysis and monitored at 407 nm for 3.50 min. The retention time (RT) of Ce6 was observed in 2.40 min. The mass spectra were recorded in TSQ vantage triple quadruple (Thermo, San Jose, CA, USA) by turbo spray ionization in the positive mode. Multiple reaction monitoring (MRM) transition was *m*/*z*: 597.2–465.308. 

### 4.7. Mouse Model

Two sets of experiments with an obesity-induced mouse model were set up for the in vivo study at Dongsung Cancer Center and In Vivo Co., Ltd. (Non-clinical CRO, Nonsan, Republic of Korea), respectively. In the first set of experiments, four-week-old male C57BL6 mice (*n* = 20, 16–18 g) were purchased from Orient Bio (Sungnam, Republic of Korea). The normal diet (10% fat) and high-fat diet (60%) were purchased from LabAnimal Co. (Seoul, Republic of Korea). The animals were housed and maintained in a standard environment (room temperature 20 ± 2 °C; humidity 50 ± 5%; light/dark cycle: 12:12 h) in the standard lab facility of Dongsung Cancer Center for a week. The mice were randomly divided into 5 groups with similar weights; normal group (Nor), vehicle-treated high-fat diet group (HFD), high-fat diet with Ce6 (2.5 mg/kg) and light of a high fluence rate (HC2.5Hi), high-fat diet with Ce6 (5 mg/kg) and light of a low fluence rate (HC5Lo), and high-fat diet with Ce6 (5 mg/kg) and light of a high fluence rate (HC5Hi) (Table 5). Before Ce6-PDT treatment, the normal group of mice was fed a normal diet (10% fat), while the remaining groups were fed a high-fat diet (60% fat) for 70 days to increase their body weight by about 70%. All the mouse experiments in the mouse model set were reviewed and carried out with the approval of the Institutional Animal Care and Use Committee of the Dongsung Cancer Center under protocol IACUC #ds0022010107-2. 

In the case of the second set of experiments, four-week-old male C57BL6 mice (*n* = 56, 16–18 g) were purchased from Samtaco Bio Korea (Osan, Republic of Korea). The normal diet (10% fat) and high-fat diet (60%) were purchased from Saeronbio, Inc. (Uiwang, Republic of Korea). Animals were housed in the standard animal house facility (room temperature 20 ± 2 °C; humidity: 50 ± 5%; light/dark cycle: 12:12 h) of the InVivo Co., Ltd. Bio Center for 7 days and were divided into 8 groups (Table 6). The normal group of mice were fed a normal diet (10% fat), while the remaining groups were fed a high-fat diet (60% fat). Before the administration of Ce6-PDT, the mouse weights increased by 30% in 42 days. All the mouse experiments in model set 2 were reviewed and carried out with the approval of the InVivo Co., Ltd. Bio Center (Non-clinical CRO, Nonsan, Republic of Korea) under approval no. IV-RB-04-1807-32.

**Table 5:** Preliminary experimental mouse model set 1.

Test Group	No. of Mice	Feeding	Amount of Test Substance
Normal group (Nor)	4	Normal diet	N.S.
Vehicle-treated HFD	4	High-fat diet	N.S.
HC2.5Hi	4		Ce6 2.5 mg/kg + High LED
HC5Lo	4		Ce6 5 mg/kg + Low LED
HC5Hi	4		Ce6 5 mg/kg + High LED

### 4.8. Ce6 Anti-obesity Efficacy Evaluation

The experiments on mouse model sets 1 and 2 were carried out according to Table 5 and Table 6. As Ce6 was found to be most concentrated at 2.5 and 5 mg/kg on sub-acute treatment in mice from a preliminary pharmacokinetics study, we selected the two doses. Ce6 (2.5 mg/kg/day or 5 mg/kg/day) was dissolved in normal saline, filtered through a 0.2 µm syringe filter, and administered orally to the treated mice of sets 1 and 2 every two days and three times a week, respectively. For the PDT, the LED (660 nm) was used as the light source. LED light of a low fluence rate (2.56 mW/cm^2^) for 5 min. was used as low irradiation dose, whereas LED light of a high fluence rate (4.96 mW/cm^2^) for 10 min. was used as high irradiation dose. PDT was performed 3 h after administration of Ce6. We decided on 3 h of administration for Ce6 based on the pharmacokinetic findings from our earlier investigations [47]. HFD groups were treated with normal saline (vehicle). The weight changes, along with food and water intake, were measured every week. The mouse models 1 and 2 were euthanized on the 99th and 70th days, respectively, by cervical dislocation.

### 4.9. Determination of Leptin and Adiponectin by ELISA

To measure the leptin and adiponectin contents of the obese mouse, all the mice were fasted for 12 h before the final day of sacrifice. The plasma levels of leptin were determined using blood samples using the Leptin Mouse ELISA Kit (ab100718, Abcam, Boston, MA, USA) following the manufacturer’s instructions. Plasma adiponectin was determined using the Mouse Adiponectin/Acrp30 Kit (R&D Systems Inc., a Bio-Techne Brand, Minneapolis, MN, USA) as described by the manufacturer. Both samples were measured on a microplate reader at 450 nm.

### 4.10. Serum Biochemistry

Blood was collected from the mice’s vena cava 48 days after euthanization. Samples were placed on clot activator serum tubes and allowed to clot for 10–15 min. before being subjected to centrifugation (15,000 rpm for 10 min). The separated serum was further used to determine the levels of aspartate aminotransferase (AST), alanine aminotransferase (ALT), blood urea nitrogen (BUN), creatinine, total cholesterol (TC), triglycerides (TG), low-density lipoprotein (LDL), and high-density lipoprotein (HDL). Serum AST, ALT, BUN, creatinine, TC, TG, serum HDL, and LDL levels were measured using an automated hematology analyzer (TBA-120FR).

### 4.11. Development of LED Mouse Chamber

**Figure 7:**
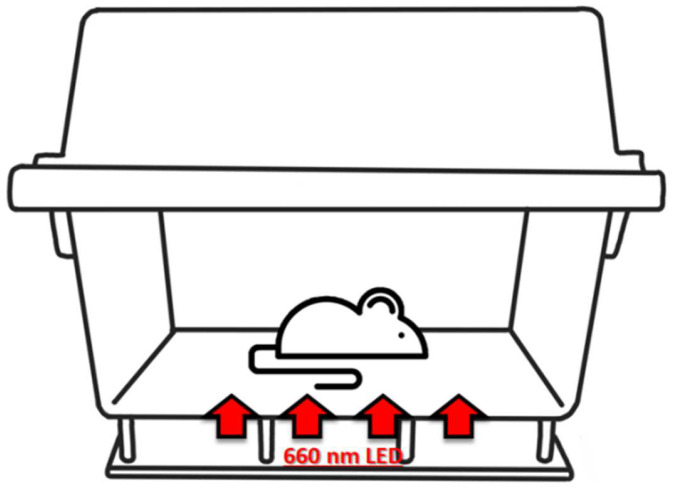
Scratch figure of LED mouse chamber.

The obesity LED package was manufactured in Lumax Co., Ltd. (Asan, Republic of Korea). The specification of the LED package was 20 mA, 2.0 V, 0.04 W, 660 nm, 35 × 28 cm size. The chip size was 330 × 330 µm^2^ ± 25 µm^2^ (thickness: 100 µm ± 10 µm, chip manufacturer/model extinction: Arima Optoelectronics, Taiwan/AOC-712RDM-Au). The LED light (96 pieces) was soldered on the printed circuit board (PCB) at a 20 × 20 mm^2^ distance between each piece. The LED circuit configuration was 12 s 8P, and the total power consumption was 4 W. Later, the LED containing PCB was fixed under the base plate of the acrylic mouse container (380 × 230 × 3 mm^3^) by using a supporter (PC material) at 40 mm height (Figure 7).

### 4.12. Statistical Analysis

All values were expressed as mean ± standard error (Mean ± SD). Statistical analysis was performed with a one-way ANOVA (one-way analysis of variance test) followed by Tukey’s multiple comparisons. A value of *p* < 0.05 was considered statistically significant.

## 5. Conclusions

In conclusion, oral administration of Ce6 with PDT for 8 weeks efficiently decreased HFD-induced body weight, adipocyte size, and increased serum HDL cholesterol in mice. Particularly, the groups HC2.5Hi, which provided 2.5 mg/kg Ce6 with a high irradiation dose, and HC5Hi, (Ce6 (5 mg/kg) with a high irradiation dose, showed the best anti-obesity activity through improved leptin, adiponectin, and serum lipid levels in high-fat diet-induced obesity in mice. Therefore, Ce6-PDT (2.5 mg/kg and 5 mg/kg of Ce6 with high irradiation dose) can be an effective and safe regimen to reduce obesity and prevent metabolic disorders related to obesity. Furthermore, the molecular mechanisms underlying the anti-obesity effects of Ce6-mediated PDT should be explored.

## Figures and Tables

**Table 1:** Ce6 concentration in serum of normal mouse, normal rat, and fat mouse with time.

Observed Time (h)	Calculated Serum Ce6 Concentration (ng/mL)		
	Mouse	Rat	Fat Mouse
0	ND	ND	ND
0.25	BQL	2.0	6.8
0.5	16,685.4	2.2	ND
1	12.8	11.4	7.2
2	BQL	49.1	12.2
4	67.6	241.4	129.9
6	ND	29.8	29.3
8	51.2	162.6	28.7

ND: Not detected; BQL: Below quantification level.

**Table 2:** Liver microsomal stability of Ce6 in dog and rat.

Compounds	Dog (%)	Rat (%)
Ce6	85.4	99.6
Verapamil	-	9.2

>90%: Very stable compound with a half-life of more than 3 h; 70~90%: Stable compound with a half-life of 1~3 h; 50~70%: Relatively stable compound with a half-life from 30 to 60 min; 30~50%: Relatively unstable compound with a half-life from 15 to 30 min; <30%: Unstable, rapidly metabolized compound with a half-life of less than 15 min.

**Table 3:** Plasma stability of Ce6 in dog and rat.

Compounds	Rat (% Remaining)	
	30 min	120 min
Ce6	42.0	45.3
Procaine	76.2	45.1
Enalapril	26.2	<1

**Table 4:** Inhibitory activities of Ce6 in CYP isoenzymes.

Compounds	CYP Inhibitory Activity (% of Control)				
	CYP1A2	CYP2C9	CYP2C19	CYP2D6	CYP3A4
Ce6	66.9	84.1	70.3	90.1	81.9
Ketoconazole	89.1	91.1	95.4	97.7	24.4

Potent inhibition: IC50 < 1 µM; Moderate inhibition: 1 µM < IC50 < 10 µM; No or weak inhibition: IC50 > 10 µM.

**Table 6:** Experimental mouse model set 2.

Test Group	No. of Mice	Feeding	Amount of Test Substance
Normal group (Nor)	8	Normal diet	N.S.
Control 1—Vehicle-treated HFD	8	High-fat diet	N.S.
Control 2—HC2.5	8		Ce6 2.5 mg/kg only
Control 3—HL	8		Low LED only
Low-dose group 1—HC2.5Lo	8		Ce6 2.5 mg/kg + Low LED
Low-dose group 2—HC2.5Hi	8		Ce6 2.5 mg/kg + High LED
High-dose group 1—HC5Lo	8		Ce6 5 mg/kg + Low LED
High-dose group 2—HC5Hi	8		Ce6 5 mg/kg + High LED

## Data Availability

All the data are contained within the manuscript and Supplementary Materials.

## Supplementary Materials

The following supporting information can be downloaded at: https://www.mdpi.com/article/10.3390/ph16071053/s1; Figure S1: Food and water intake of mouse model set 2; Figure S2: Effects of Ce6-PDT on serum levels of liver and kidney toxicity markers mouse model 2; Table S1: Liver tissue slide scoring criteria; Table S2: Summary of main features of Model 1 and Model 2.
