# Supplementary material for: Anti-Obesity Effect of Chlorin e6-Mediated Photodynamic Therapy on Mice with High-Fat-Diet-Induced Obesity
Source: Pharmaceuticals (Basel). 2023 Jul 24;16(7):1053. doi: 10.3390/ph16071053 (PMC10384435; doi:10.3390/ph16071053)
Supplement: Supplementary file 1 [file pharmaceuticals-16-01053-s001.zip › pharmaceuticals-2348074-supplementary.pdf]

Supplementary information

Anti-Obesity Effect of Chlorin e6-Mediated Photodynamic Therapy in High Fat Diet-Induced Obese Mice

Rajeev Shrestha[1], Pallavi Gurung[1], Junmo Lim[1], Til Bahadur Thapa Magar[1], Cheong-Wun Kim[1], Hak-Yong Lee[2], and Yong-Wan Kim[1,*]

[1]Dongsung Cancer Center, Dongsung Biopharmaceutical, Daegu 41061, Republic of Korea

[2]INVIVO Co., Ltd., Nonsan 32992, Republic of Korea

*Correspondence: thomas06@hanmail.net

TABLE OF CONTENTS

S1. Food and water intake of mouse model set 2

S2. Effects of Ce6-PDT on serum levels of liver and kidney toxicity markers mouse model

S3. Liver tissue slide scoring criteria

S4. Summary of main features of Model 1 and Model 2

S1. Food and water intake of mouse model set 2

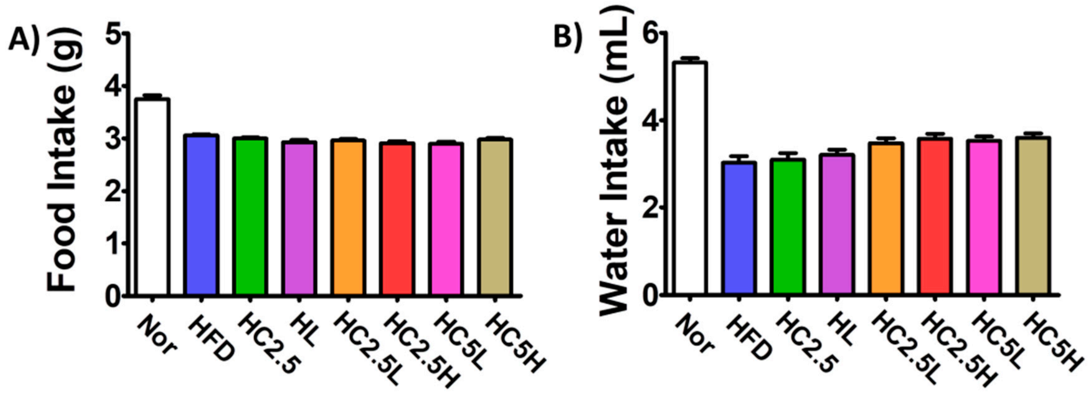

Figure S1: (A) Food and (B) water intake of mouse model set 2. Food and water were freely consumed in each test group. The consumed amount of food and water were measured by initial amount.

S2. Effect of Ce6-PDT in liver and kidney toxicity markers (AST/ALT, BUN, Creatinine)

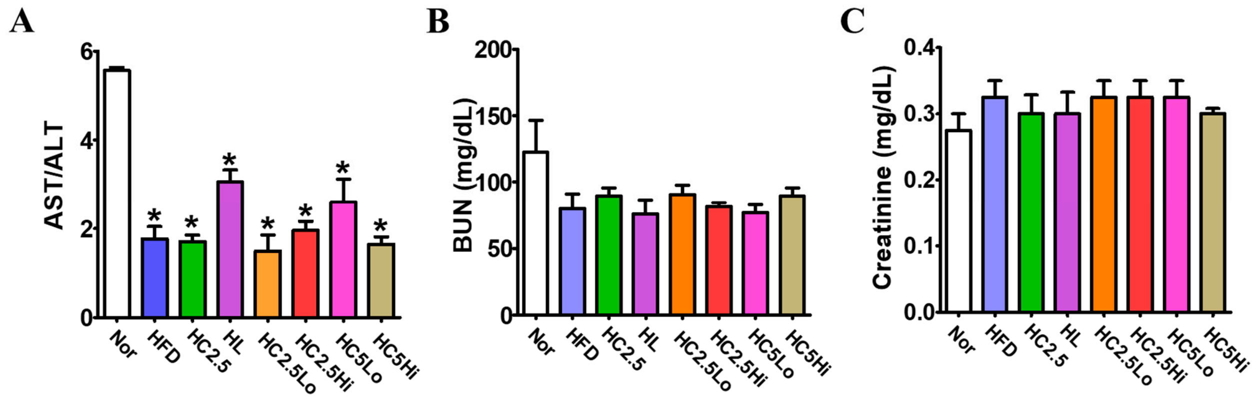

Figure S2: Effects of Ce6-PDT on serum levels of liver and kidney toxicity markers of mouse model 2. A) AST/ALT ratio, B) BUN, and C) Creatinine of the experimental mice groups. Data are expressed as mean ± SD, ($n = 3$), *$p < 0.05$, compared with the control group. #$p < 0.05$, compared with the HFD only-treated group.

S3. Liver tissue slide scoring criteria

Table S1: Liver tissue slide scoring criteria

Histological criteria	Description	Score
Steatosis	< 5%	0
	5~30%	1
	> 33~66%	2
	> 66%	3
Lobular Inflammation	No foci	0
	< 2 foci per 200× field	1
	2~4 foci per 200× field	2
	> 4 foci per 200× field	3
Hepatocellular Hypertrophy	< 5%	0
	5~30%	1
	> 33~66%	2
	> 66%	3

S4. Summary of main features of Model 1 and Model 2

Table S2: Major features of Model 1 and Model 2

S.N.	Features	Model 1	Model 2
1	Purpose	Preliminary experiment	Main experiment
2	Experimental Site	Conventional animal facility	CRO for animal experiment
3	Groups	Normal, Vehicle-treated HFD, HC2.5Hi, HC5Lo and HC5H	Normal, Vehicle-treated HFD, HC2.5, HL, HC2.5Lo, HC2.5Hi, HC5Lo and HC5Hi
4	No of mice in each group	4	8
5	Age of mice	4 weeks	4 weeks
6	Fat (%) in diet	10% in NFD and 60% in HFD	10% in NFD and 60% in HFD
7	Length of HFD exposure	70 days	42 days
8	% Increase in Fat	70%	30%
9	Doses of Ce6	2.5 and 5 mg/kg	2.5 and 5 mg/kg
10	Route of administration of Ce6	Oral	Oral
11	Fluence of LED Light	2.56 mW/cm^2 for 5 min and 4.96 mW/cm^2 for 10 min	2.56 mW/cm^2 for 5 min and 4.96 mW/cm^2 for 10 min
12	Days of PDT treatment	Every 2 days up to 29 days	Every 2 days up to 29 days
13	(%) Effectiveness of HC2.5Hi and HC5Hi groups compared to HFD group	10%	14%
